# Biocompatible MXene (Ti_3_C_2_T_x_) Immobilized with Flavin Adenine Dinucleotide as an Electrochemical Transducer for Hydrogen Peroxide Detection in Ovarian Cancer Cell Lines

**DOI:** 10.3390/mi12080862

**Published:** 2021-07-22

**Authors:** Ramila D. Nagarajan, Preethika Murugan, Kanagaraj Palaniyandi, Raji Atchudan, Ashok K. Sundramoorthy

**Affiliations:** 1 Department of Chemistry, SRM Institute of Science and Technology, Kattankulathur 603 203, Tamil Nadu, India; ramiladevi15@gmail.com (R.D.N.); preethikam95@gmail.com (P.M.); 2 Department of Biotechnology, SRM Institute of Science and Technology, School of Bioengineering, Kattankulathur 603 203, Tamil Nadu, India; kanagarp@srmist.edu.in; 3 School of Chemical Engineering, Yeungnam University, Gyeongsan 38541, Korea; atchudanr@yu.ac.kr

**Keywords:** FAD/Ti_3_C_2_T_x_, charge transfer process, sensing of H_2_O_2_, real time application, cancer cell lines

## Abstract

Flavin adenine dinucleotide (FAD) is a coenzyme and acts as a redox cofactor in metabolic process. Owing to such problems as poor electron transfer properties, unfavorable adsorption, and lack of stability on rigid electrodes, the bio-electrochemical applications of FAD have been limited. Herein, a novel fabrication method was developed for the immobilization process using 2D MXene (Ti_3_C_2_T_x_), which enhanced the redox property of FAD and improved the electro-catalytic reduction of hydrogen peroxide (H_2_O_2_) in neutral medium. The FAD-immobilized Ti_3_C_2_T_x_ electrode (FAD/Ti_3_C_2_T_x_) was studied by UV-Visible and Raman spectroscopies, which confirmed the successful adsorption of FAD on the Ti_3_C_2_T_x_ surface. The surface morphology and the elemental composition of Ti_3_C_2_T_x_ were investigated by high resolution transmission electron microscopy and the energy dispersive X-ray analysis. The redox property of the FAD/Ti_3_C_2_T_x_ modified glassy carbon electrode (FAD/Ti_3_C_2_T_x_/GCE) was highly dependent on pH and exhibited a stable redox peak at −0.455 V in neutral medium. Higher amounts of FAD molecules were loaded onto the 2D MXene (Ti_3_C_2_T_x_)-modified electrode, which was two times higher than the values in the reported work, and the surface coverage (ᴦ_FAD_) was 0.8 × 10^−10^ mol/cm^2^. The FAD/Ti_3_C_2_T_x_ modified sensor showed the electrocatalytic reduction of H_2_O_2_ at −0.47 V, which was 130 mV lower than the bare electrode. The FAD/Ti_3_C_2_T_x_/GCE sensor showed a linear detection of H_2_O_2_ from 5 nM to 2 µM. The optimization of FAD deposition, amount of Ti_3_C_2_T_x_ loading, effect of pH and the interference study with common biochemicals such as glucose, lactose, dopamine (DA), potassium chloride (KCl), ascorbic acid (AA), amino acids, uric acid (UA), oxalic acid (OA), sodium chloride (NaCl) and acetaminophen (PA) have been carried out. The FAD/Ti_3_C_2_T_x_/GCE showed high selectivity and reproducibility. Finally, the FAD/Ti_3_C_2_T_x_ modified electrode was successfully applied to detect H_2_O_2_ in ovarian cancer cell lines.

## 1. Introduction

**Scheme 1:**
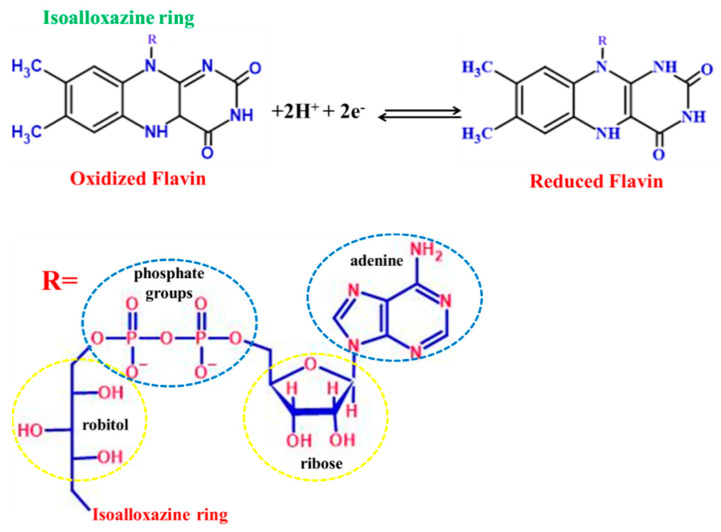
Illustration of the redox mechanism of FAD.

Inspired by nature, the mimicking of naturally occurring enzymes and the study of bio-electron transfer reactions using in-vitro models has been the long term goal of researchers [1]. The detection of hydrogen peroxide (H_2_O_2_) is of great importance in the fields of food industry, organic reactions, cell signaling and clinical systems [2]. The artificial enzymes have been used to understand physiological electron transfer reactions and for the development of bio-electrodes [3,4]. In this connection, the biomolecules gained immense interest in relation to the fabrication of catalytic material for the reduction of H_2_O_2_ [5]. Flavin mononucleotide (FMN) and flavin adenine dinucleotide (FAD) are the cofactors of riboflavin, which is known as vitamin B2 [6]. The flavin cofactors contain the isoalloxazine ring, which tethered to the ribityl phosphate or ribityl adenine diphosphate chain [7,8]. These flavin proteins are involved in a wide range of biochemical reactions involving the transferring of two or single electrons in a single step or two-step process, as shown in Scheme 1. The flavin proteins are attracted towards the biological system where it acts as a photo receptor [9]. The FAD is composed of a unique structure with isoalloxazine ring attributes the fluorescence, and the attached adenine moiety as the quencher. The structure of FAD exists in three conformations (stacked, unstacked and partially stacked) [10]. Depending on the pH, FAD can show different conformations, exhibit fluorescence properties, and its lifespan also varies [11]. Due to FAD’s peculiar properties, the vast applications of FAD were demonstrated in imaging and catalysis reactions [12]. Moreover, due to its simple electron transfer property, different FAD-modified electrodes had been demonstrated for various electrochemical sensor applications [13]. For example, FAD immobilized on nickel oxide-modified GCE was used for the detection of persulphate from 3 µM to 1.5 mM and the limit of detection (LOD) was 0.38 µM [14]. FAD/cobalt oxide nanoparticle-immobilized GCE was also utilized for the detection of nitrite from 1 to 30 µM with a limit of detection(LOD) of 0.2 µM [15].

However, the direct immobilization of FAD on bare GCE and other noble metal electrodes were found to be difficult due to its large size and lack of stability [14]. These issues had been avoided by using humic acid/halloysite nanotube(HA/HNT) modified electrodes [16], carbon nanotube/chitosan electrodes [17] and electrochemically deposited titanium oxide nanoparticles (TiO_2_) [18]. Thus, the successfully immobilized FAD on TiO_2_ modified GCE showed a redox peak at −0.45 V [18]. In order to further improve the immobilization process and electrocatalytic applications of FAD, a new generation of 2D materials have been used. Recently, 2D transition metal carbides, nitrides and carbonitrides have been synthesized as MXenes [19,20]. M_n+1_X_n_T*_X_* is the general formula of MXene, where M stands for the metal and X is for nitrogen, carbon and carbonitrides and T is the functional groups (hydroxyl, oxygen and fluorine) on the surface [21,22]. Among the other 2D materials, MXene exhibited some special properties such as hydrophilicity, high specific surface area and good adsorption phenomena [23]. Due to the peculiar properties of MXenes, they have been used in various applications which include supercapacitors [24], batteries [25], microbial fuel cells [26], photocatalysis [27] and sensors [19]. Recently, MXene based electrochemical sensors had been reported for the sensing of glucose [28], ascorbic acid (AA), dopamine (DA) and uric acid (UA) [29], L-cysteine [30] and superoxide anions from human liver cancer cell lines (HepG2) [31]. In addition, enzymatic [32] and a non-enzymatic H_2_O_2_ sensors were also prepared using the platinum nanoparticle (Pt NPs) decorated MXene [33] and polyaniline/Pt NPs/MXene modified electrodes [34].

In this work, for the first time, we reported MXene as a host material for the immobilization of FAD and used it as a H_2_O_2_ biosensor. Firstly, the Ti_3_C_2_T_x_ (MXene) modified electrode was prepared and immobilization of FAD was carried out by electrochemical deposition. The surface coverage of FAD was higher on Ti_3_C_2_T_x_ than the reported methods. Secondly, the selectivity of the FAD/Ti_3_C_2_T_x_ modified electrode was investigated against peroxide reduction. Finally, the real sample analysis of the FAD/Ti_3_C_2_T_x_ electrode was tested by detecting spiked H_2_O_2_ in the sample with cancer cell lines.

## 2. Materials and Methods

### 2.1. Materials

Preparation of Ti_3_C_2_T_x_ was carried out as reported elsewhere [29]. FAD and DA were purchased from the Sigma-Aldrich, Bangalore India. The other used chemicals, such as AA, UA, OA, PA, glucose, lactose, NaCl, KCl, monosodium dihydrogen phosphate (NaH_2_PO_4_), disodium hydrogen phosphate (Na_2_HPO_4_), L-cystine, L-isoleucine, L-alanine and L-tyrosine, were obtained from SRL chemicals, Chennai, Tamil Nadu, India. The phosphate buffer solution (PBS) (supporting electrolyte) and the different pH solutions were prepared using (NaH_2_PO_4_ and Na_2_HPO_4_). OVCAR-5 and SKOV-3 ovarian cancer cell lines were procured from ATCC, Manassas, VA, USA. RPMI-1640 medium, fetal bovine serum (FBS), trypsin EDTA and antibiotics were obtained from Sigma-Aldrich, St. Louis, Missouri, MO, USA.

### 2.2. Apparatus

Electrochemical studies were carried out on CH Instruments—760E (CH Instruments, Austin, TX, USA) using a three-electrode system with FAD/Ti_3_C_2_T_x_ or unmodified GCE as the working electrode. The Ag/AgCl/3M KCl and platinum wire were used as the reference and the counter electrodes. For the electro-catalytic study, the de-aerated supporting electrolyte was used. The physiochemical characterization of materials, and the charge transfer properties of FAD/Ti_3_C_2_T_x_, were analyzed using UV-visible spectroscopy—2000c Nanodrop technology, Wilmington, NC, USA. The Raman spectrum of FAD/Ti_3_C_2_T_x_ was recorded with the 532 nm laser excitation source, using a Micro-Raman spectrometer (Labram HR evolution-Horiba, Eugène Avinée, France). The morphology and elemental composition of Ti_3_C_2_T_x_ were analyzed by high resolution transmission electron microscopy (HR-TEM) and energy dispersive X-ray (EDX) analysis, using a 2100 plus Electron microscope (JEOL, Tokyo, Japan).

### 2.3. Preparation of FAD/Ti_3_C_2_T_x_ Modified Sensor

**Scheme 2:**
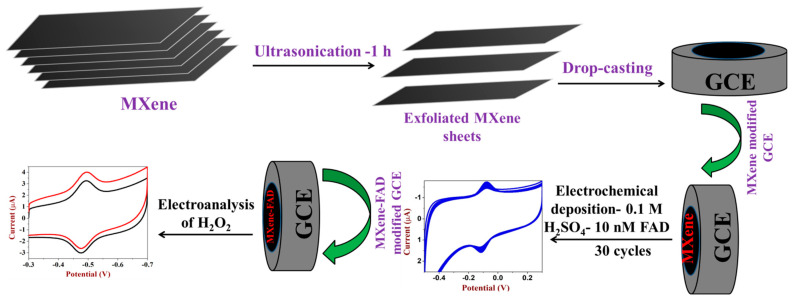
Electro-deposition of FAD (from 0.1 M H_2_SO_4_ containing 10 nM FAD) on to biocompatible Ti_3_C_2_T_x_ modified GCE for the electro-catalytic reduction of H_2_O_2_.

To prepare Ti_3_C_2_T_x_ dispersion, different amounts of Ti_3_C_2_T_x_ (0.2, 0.4, 0.6, 0.8 and 1 mg/mL) powder were probe sonicated in distilled water for 1 h with the amplitude of 54%, 3 s ON and 2 s OFF. Then, the each one of the Ti_3_C_2_T_x_ dispersions (10 µL) was coated on the GCE and dried at 50 °C for 5 min. For the process of FAD immobilization, the Ti_3_C_2_T_x_ modified electrode was used as shown in Scheme 2. The FAD immobilization was successfully carried out using a Ti_3_C_2_T_x_ modified electrode by potential sweeping between 0.3 and −0.5 V for 30 continuous CV cycles in 0.1 M H_2_SO_4_ containing 10 nM FAD. After that, the FAD-immobilized Ti_3_C_2_T_x_ electrode was completely dried at room temperature for 30 min. The FAD/Ti_3_C_2_T_x_ modified electrode showed a redox peak at −0.1 V in acidic medium [18]. However, when a FAD/Ti_3_C_2_T_x_ electrode was used to record cyclic voltammograms (CVs) in 0.1 M PBS, it showed a redox peak at −0.455 V due to the pH effect. Moreover, FAD/Ti_3_C_2_T_x_ modified GCE was applied for the electro-analysis of H_2_O_2_ in de-aerated PBS solution. The FAD/Ti_3_C_2_T_x_ modified electrode was stored in PBS when not in use (Scheme 2).

### 2.4. Real Sample Preparation

OVCAR-5 and SKOV-3 cell lines were cultured in RPMI-1640 medium supplemented with 10% FBS, 1% penicillin and streptomycin. The cells were maintained under 5% CO_2_ atmospheric pressure at 37 °C in a CO_2_ incubator. The cells were sub-cultured using Trypin (0.05%) and EDTA (0.02%). The trypsinized cells were washed with 10% FBS media to inhibit trypsin activity and further washed twice with PBS (0.1 M). The cell viability was determined by using Trypan Blue exclusion method. The viable cells (1 × 10^6^/mL) were re-suspended separately and used for electro-analysis of H_2_O_2_.

## 3. Results and Discussion

### 3.1. UV-Vis and Raman Spectroscopy

**Figure 1:**
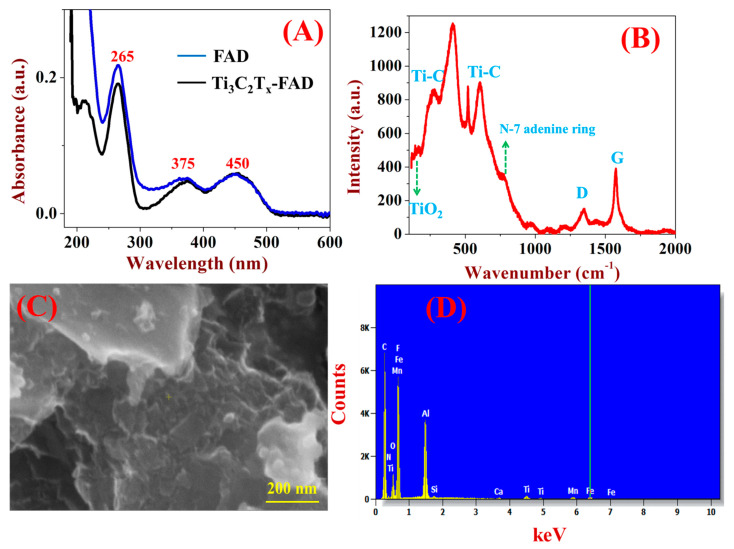
(**A**) UV-Vis spectra of the FAD and Ti_3_C_2_T_x_-FAD solutions. (**B**) The Raman spectrum of the FAD/Ti_3_C_2_T_x_ film. (**C**) The HR-TEM image and (**D**) EDX spectrum of Ti_3_C_2_T_x_.

The interaction between FAD and Ti_3_C_2_T_x_ was analyzed by UV-Vis spectroscopy (UV-Vis). The UV-Vis absorption spectra of FAD showed three transition peaks at 265, 375 and 450 nm. The absorption bands at 375 and 450 nm corresponded to the oxidized form of FAD (Figure 1A). The absorption at 375 nm was due to the S_0_–S_2_ transition and 450 nm peak for the S_0_–S_1_ transition [35]. To investigate the charge-transfer between MXene and FAD, 0.3 mL of (1 mg/mL) Ti_3_C_2_T_x_ dispersion was added to the 100 nM FAD aqueous solution and mechanically mixed well using an orbital shaker. The FAD solution showed an absorbance peak, with high intensity, due to isoalloxazine. However, after FAD interacted with MXene, the absorbance peak of FAD decreased slightly, which may be due to the interaction of FAD with Ti_3_C_2_T_x_ through the surface functional groups (Figure 1A). From the UV-Vis spectra, it was confirmed that the N-7 adenine group might have interacted with the Ti_3_C_2_T_x_ [14].

The Raman spectrum of FAD/Ti_3_C_2_T_x_ was recorded using the 532 nm laser excitation. As reported earlier, the Raman spectrum of the Ti_3_C_2_T_x_ showed the presence of Ti-C, Ti-Al and TiO_2_ vibrational bands [36]. The major Ti_3_C_2_T_x_ vibration bands appeared at 215, 352, 620 and 688 cm^−1^ [29], which confirmed the formation of Ti_3_C_2_T_x_. The Ti-C vibrational bands appeared at 352 [29], 620 and 688 cm^−1^ [37]. TiO_2_ vibration was also observed at 157 cm^−1^. Additionally, the Ti-Al vibration band was found at 215 cm^−1^ which indicated the formation of mixed phase of MXene [36]. The functional groups present on the Ti_3_C_2_T_x_ were helped in the strong immobilization of FAD by electrochemical deposition. During the deposition of FAD on Ti_3_C_2_T_x_, the (N-7) adenine ring of FAD may be oriented in parallel with the Ti_3_C_2_T_x_ surface, as was confirmed by the vibrational band of adenine found at 775 cm^−1^ [38]. In addition, for the FAD/Ti_3_C_2_T_x_ film, TiO_2_ and Ti-C vibrational bands were shifted to higher wavenumbers at 168 and 271 cm^−1^ (Figure 1B). Moreover, the graphitic carbon bands appeared at 1347 (D band) and 1577 cm^−1^ (G band), which corresponded to the sp^3^ and sp^2^ hybridized carbon atoms. The calculated ratio of I_D_/I_G_ was 0.4, which indicated that there were less defects present on the surface of FAD/Ti_3_C_2_T_x_ than the individual Ti_3_C_2_T_x_ (I_D_/I_G_ = 1.8).

The surface morphology of Ti_3_C_2_T_x_ was recorded by HR-TEM, which confirmed that the layered/sheet-like morphology was obtained and the average sheet sizes were about 400–500 nm (Figure 1C). The d-spacing of the Ti_3_C_2_T_x_ film was measured as 0.354 nm, which was matched with the d value of Ti-C. Furthermore, the elemental mapping analysis also showed the presence of the Ti, Al, C, F and O elements, as shown in (Figure 1D) [29].

### 3.2. Electrochemical Characterization of FAD/Ti_3_C_2_T_x_ Modified Electrode

**Figure 2:**
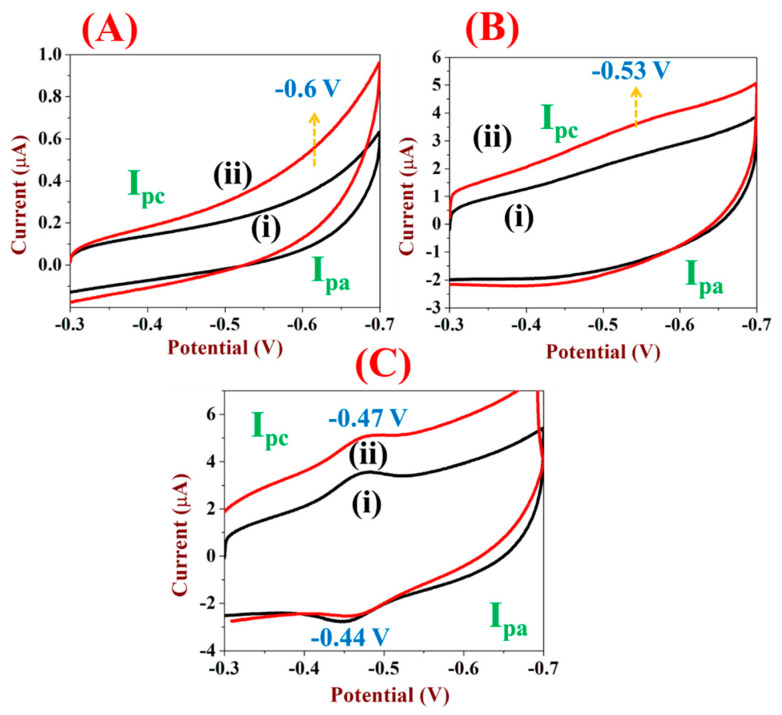
CVs were recorded using (**A**) bare GCE, (**B**) Ti_3_C_2_T_x_/GCE, and (**C**) FAD/Ti_3_C_2_T_x_/GCE in 0.1 PBS without (curves i) and with 100 nM H_2_O_2_ (curves ii) in 0.1 M PBS, scan rate = 50 mV/s.

Figure 2 shows the typical CVs of (A) bare GCE, (B) Ti_3_C_2_T_x_/GCE and (C) FAD/Ti_3_C_2_T_x_/GCE in N_2_ saturated 0.1 M PBS. The CVs of Ti_3_C_2_T_x_/GCE showed that the capacitive current increased due to intercalation of ions from the electrolyte and pseudo capacitance behavior of Ti_3_C_2_T_x_ (Figure 2B, curve i) [39]. The presence of surface functional groups on Ti_3_C_2_T_x_ might have helped to immobilize the FAD enzyme without any additional binder molecules. The redox activity of the FAD enzyme was strongly dependent on pH and the redox potential was also shifted with the pH. After the electrochemical deposition, FAD/Ti_3_C_2_T_x_/GCE was tested in 0.1 M PBS, which showed a redox peak at −0.455 V, and the cathodic and anodic peak currents appeared almost equally (Figure 2C, curve-i) [16]. It was believed that Ti_3_C_2_T_x_ film had shown greater effect on the immobilization process and transfer of electrons to the electrode surface. FAD immobilization was carried out on the bare GCE (without MXene) in the same condition. However, the FAD immobilization was found not to be favorable due to the lack of catalytic and biocompatible sites on the bare surface. The heterogeneous electron transfer kinetics of FAD were highly dependent on the catalyst material and the charge transfer interaction. The adenine ring of FAD was adsorbed on the MXene surface and the isoalloxazine ring was oriented away from the surface. The higher loading of FAD molecules on Ti_3_C_2_T_x_ surface was due to favorable interaction between them through the adenine moiety [40]. The interaction between FAD and the Ti_3_C_2_T_x_ nanosheets was also confirmed by UV-vis spectroscopy. Next, CVs were recorded for the electroanalysis of H_2_O_2_ using the (Figure 2A, curve ii) bare-GCE, (Figure 2B, curve ii) Ti_3_C_2_T_x_ and (Figure 2C, curve ii) FAD/Ti_3_C_2_T_x_/GCE’s. The bare GCE showed a broad reduction peak of H_2_O_2_ at −0.6 V. However, after modification with Ti_3_C_2_T_x_, the H_2_O_2_ reduction peak appeared at −0.53 V with the increase in catalytic current. Interestingly, at the FAD/Ti_3_C_2_T_x_/GCE (Figure 2C, curve ii), the H_2_O_2_ reduction current was increased at −0.47 V, with the reduction in the overpotential by about 130 mV as compared to the bare GCE. These results confirmed that the immobilized FAD functioned as an effective electron transfer mediator for the reduction of the H_2_O_2_ molecule to H_2_O.

### 3.3. The Effect of Ti_3_C_2_T_x_ Concentration on the FAD Immobilization

**Figure 3:**
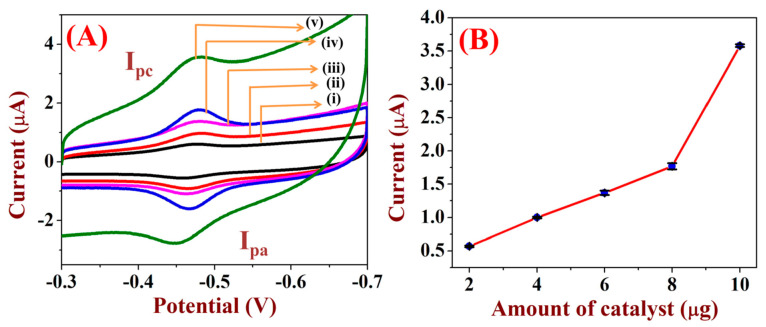
(**A**) CVs were recorded using different FAD/Ti_3_C_2_T_x_ GCE’s which were prepared with different amounts of Ti_3_C_2_T_x_: (i) 2, (ii) 4, (iii) 6, (iv) 8 and (v) 10 µg. For all the electrodes, electro-deposition of FAD was carried out using 10 nM FAD in 0.1 M H_2_SO_4_ for 30 cycles, scan rate = 50 mV/s. (**B**) The non-linear plot shows the relationship between the amount of Ti_3_C_2_T_x_ and the reduction peak currents of FAD (immobilized).

In order to find out the optimum amount of Ti_3_C_2_T_x_ for the FAD immobilization process, dispersions of different Ti_3_C_2_T_x_ concentrations (0.2, 0.4, 0.6, 0.8 and 1 mg/mL) were prepared and 10 µL of each was coated on the GCE. Each of the Ti_3_C_2_T_x_/GCE was used to immobilize FAD molecules. Figure 3A shows the CVs of FAD immobilized on different modified GCE’s with different amounts of Ti_3_C_2_T_x_ films (see Section 2.3). The obtained results showed that each of the Ti_3_C_2_T_x_/GCE had the ability to adsorb FAD molecules. However, when we varied the amount of Ti_3_C_2_T_x_ (host) on the GCE, the surface adsorption of FAD was also increased. For the lowest amount of Ti_3_C_2_T_x_, a thin layer of FAD was formed on the surface of Ti_3_C_2_T_x_ [41]. When the amount of Ti_3_C_2_T_x_ was gradually increased, the deposition of FAD (molecules) was also increased on the electrode surface. The surface coverage of FAD was calculated for the all electrodes using Equation (1) [42].

(1) Γ=QnFA

where, *Q* is the coulombic charge, *n* is number of electrons, F is faraday constant and *A* is the surface area of the electrode [43]. Using the above parameters, the ᴦ_FAD_ was calculated as 0.0681 × 10^−10^, 0.139 × 10^−10^, 0.15 × 10^−10^, 0.35 × 10^−10^, and 0.7 × 10^−10^ for 2, 4, 6, 8 and 10 µg of Ti_3_C_2_T_x_ on GCE’s. As can be seen, with the increase in the quantity of Ti_3_C_2_T_x_ on GCE, the FAD surface adsorption was also increased. Figure 3B shows the non-linear plot established between the amount of Ti_3_C_2_T_x_ on GCE and cathodic peak currents of FAD. This study demonstrated that the GCE coated with 10 µL of 1 mg/mL Ti_3_C_2_T_x_ dispersion was suitable for maximum adsorption of FAD. Due to its hydrophilic functional groups, high surface area and biocompatible nature, MXene strongly interacted with the FAD and helped in the immobilization process. Finally, 1 mg/mL (10 µL) Ti_3_C_2_T_x_ dispersion was selected for the immobilization of FAD to carry out further studies on the electrocatalytic reduction of H_2_O_2_.

### 3.4. The Optimization of FAD Loading on Ti_3_C_2_T_x_/GCE

**Figure 4:**
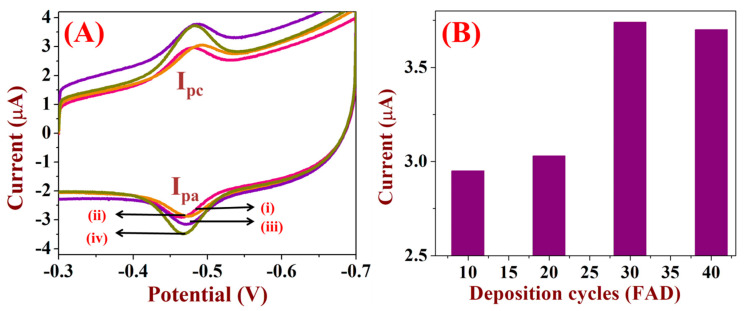
(**A**) CVs were recorded using Ti_3_C_2_T_x_/GCE after the FAD immobilization process by varying the number of potential cycles (i—10, ii—20, iii—30 and iv—40 cycles) in 0.1 M PBS, scan rate = 50 mV/s. (**B**) The bar diagram represents the relationship between the FAD deposition cycles and the reduction peak current of FAD/Ti_3_C_2_T_x_/GCE’s.

The amount of FAD immobilized on Ti_3_C_2_T_x_ may play a major role in the enhancement of the H_2_O_2_ reduction current. To tune the optimum FAD loading on Ti_3_C_2_T_x_/GCE for the effective H_2_O_2_ reduction, 10 µL of (1 mg/mL) Ti_3_C_2_T_x_ dispersion was drop-casted on the GCE and used for the immobilization of FAD (see Section 2.3). The immobilization of the FAD process was carried out from 0.1 M H_2_SO_4_ containing 10 nM FAD by varying the potential cycles between 0.3 and −0.5 V (examples: 10, 20, 30 and 40 cycles). After that, CVs of each FAD/Ti_3_C_2_T_x_/GCE were recorded in 0.1 M PBS, as shown in Figure 4A. The lowest peak currents (low adsorption) of FAD were recorded for the electro-deposition of 10 cycles. With the number of potential cycles increased, FAD adsorption was also increased, and the maximum adsorption of FAD was recorded for 30 cycles. Figure 4B illustrates the relationship between the FAD reduction peak current vs. number of electrochemical deposition cycles. After 30 CV cycles, the FAD adsorption (immobilization) was saturated, which indicated that the FAD was entirely adsorbed on the Ti_3_C_2_T_x_/GCE surface. From this study, 1 mg/mL (10 µL) Ti_3_C_2_T_x_ dispersion was found to be optimal for the modification of the GCE surface to immobilize higher amounts of FAD through 30 potential cycles in 0.1 M H_2_SO_4_ containing 10 nM FAD. As a control experiment, FAD adsorption was carried out on bare GCE (without MXene); it did not help to immobilize FAD molecules. So, it was confirmed that Ti_3_C_2_T_x_ might have played a major role in the immobilization process of FAD.

### 3.5. Kinetic Study of FAD/Ti_3_C_2_T_x_/GCE

**Figure 5:**
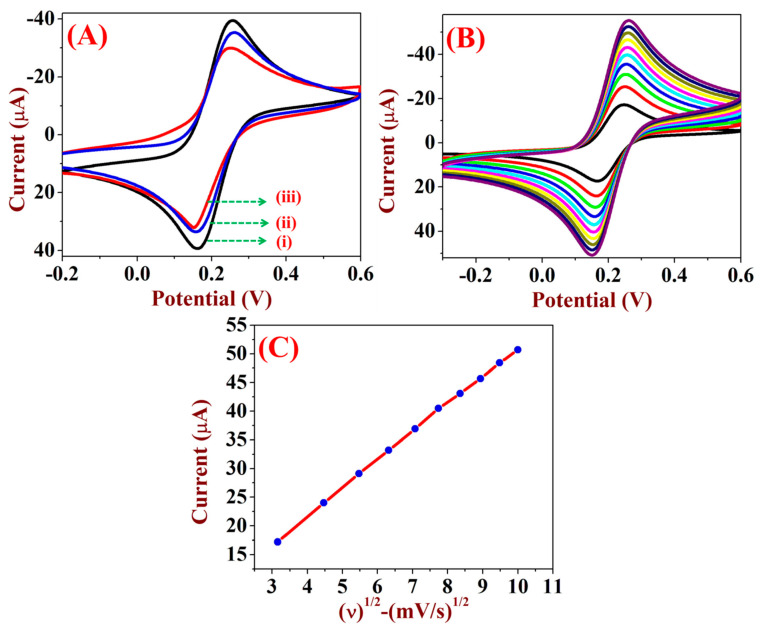
(**A**) CVs were recorded using unmodified electrode (curve i), FAD/Ti_3_C_2_T_x_/GCE (curve ii) and Ti_3_C_2_T_x_/GCE (curve iii) in 0.1 M KCl/5 mM [Fe(CN)_6_]^3−/4−^ at the scan rate of 50 mV/s. (**B**) CVs for FAD/Ti_3_C_2_T_x_/GCE at different scan rates from 10 to 100 mV/s. (**C**) The linear plot was made between the *ν*^1/2^ and the reduction peak currents.

The electrode kinetics of the FAD/Ti_3_C_2_T_x_ modified electrode were studied in 0.1 M KCl with 5 mM [Fe(CN)_6_]^4−/3−^ (Figure 5A). For the bare electrode, the peak-to-peak separation (ΔE_p_) was about 120 mV with the peak current of ~40 µA (curve i). After modification with Ti_3_C_2_T_x_, the ΔE_p_ value was shifted to 100 mV with the decrease in peak current (29.89 µA). Ti_3_C_2_T_x_ acts as the electron transfer mediator between the electrode and the electrolyte but the redox peak currents were decreased due to the negatively charged repulsion between the Ti_3_C_2_T_x_ electrode and [Fe(CN)_6_]^4−/3−^ (curve iii) [44]. Furthermore, after FAD immobilization on Ti_3_C_2_T_x_, the redox peak currents of 5 mM [Fe(CN)_6_]^4−/3−^ were increased on the FAD/Ti_3_C_2_T_x_ modified electrode as compared to the Ti_3_C_2_T_x_/GCE (curve ii). From this comparative CVs, it was found that FAD/Ti_3_C_2_T_x_/GCE possess good electron conduction pathways compared to the other modified electrodes [18]. Next, CVs were recorded using FAD/Ti_3_C_2_T_x_/GCE at varying the scan rates from 10 to 100 mV/s (Figure 5B). This study helped to examine whether the electrode reaction was a diffusion-controlled or surface-controlled process. Figure 5C shows the linear plot was drawn for the square root of the scan rate (*ν*)^1/2^ *vs.* I_pc_ (cathodic peak current of 5 mM [Fe(CN)_6_]^3−/4−^). This data indicated that the peak current (anodic and cathodic) increases linearly with respect to (*ν*)^1/2^, and the linear regression equation was Y = 4.88 × 10^−6^ + 2.154 × 10^−6^ (R^2^ = 0.999). This was due to the diffusion-controlled process at lower scan rate [45]. From the slope value of the linear curve, the surface area (A) of the different electrodes were calculated using the Randles–Sevcik Equation (2) [46]. The parameters are as follows: ‘n’ is the number of electrons (*n* = 1), D is the diffusion coefficient of [Fe(CN)_6_]^3−/4−^] (7.6 × 10^−6^ cm^2^/s), *C_o_* is the concentration of [Fe(CN)_6_]^3−/4−^ (5 mM) and A is the surface area of the electrode.

(2) $$I_{pa}=\left(2.69\times 10^{5}\right)n^{\frac{3}{2}}D^{\frac{1}{2}}C_{o}\nu^{\frac{1}{2}}A$$

Using Equation (2), the electrochemical surface area (A) was calculated for FAD/Ti_3_C_2_T_x_/GCE, bare GCE and Ti_3_C_2_T_x_/GCE as 0.13 cm^2^, 0.113 cm^2^ and 0.078 cm^2^, respectively. It was found that the surface area was higher for FAD/Ti_3_C_2_T_x_/GCE compared to the other modified electrode and bare GCE.

### 3.6. The Effect of PH

**Figure 6:**
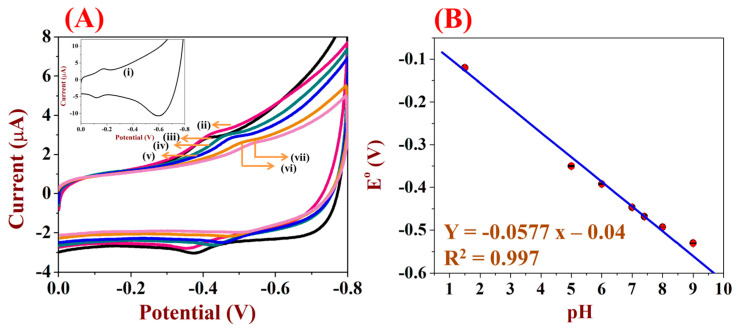
(**A**) CVs were recorded using FAD/Ti_3_C_2_T_x_/GCE in different pH buffers [(ii) pH 5, (iii) pH 6, (iv) pH 7, (v) pH 7.4, (vi) pH 8 and (viii) pH 9]. Inset figure was the CVs of FAD/Ti_3_C_2_T_x_/GCE recorded at pH 1.5 (curve i) at the scan rate of 50 mV/s. (**B**) The linear plot was made between the pH and formal potentials of FAD.

The pH effect on the redox activity of FAD/Ti_3_C_2_T_x_/GCE was studied in different pH solutions (examples: pH 1.5, 5, 6, 7, 7.4, 8, and 9). It was already known that the redox peak of FAD was highly dependent on the pH. Since the redox behavior of FAD was due to the presence of isoalloxazine ring, the CVs of the FAD/Ti_3_C_2_T_x_/GCE were shifted with the pH of the buffer solutions. As shown in Figure 6A, the redox peak was highly active in acidic medium due to the structural conformation of FAD. In acidic solution, FAD was present in the open stack structure [47]. When the pH of the solution was increased, the FAD structural conformation changed to a closed structure at high pH [12]. The formal potential of FAD was −0.1 V in acidic medium; when the pH of solution was increased, the formal potential was shifted to more negative values and the peak current started to decline (Figure 6A). A linear plot was made between the pH and formal potentials, as shown in Figure 6B, which gave a linear regression equation with the slope value of −57.7 mV/pH. This slope value was closer to −59 mV/pH, which indicated that the transfer process of FAD, with equal numbers of protons and electrons, showed Nernstian behavior [48]. From this study, neutral pH (0.1 M PBS, pH = 7.4) was selected for the further analysis of H_2_O_2_ in in-vivo studies.

### 3.7. Effect of Scan Rate

**Figure 7:**
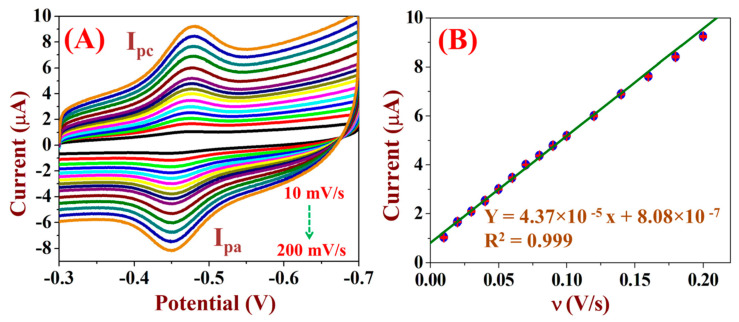
(**A**) CVs were recorded using FAD/Ti_3_C_2_T_x_/GCE in 0.1 M PBS at different scan rates from 0.01 to 0.2 V/s. (**B**) The linear plot was made between the scan rates and the reduction peak currents.

Figure 7A illustrates the CVs of FAD/Ti_3_C_2_T_x_ modified GCE recorded at different scan rates from 0.01 to 0.2 V/s. The redox peak currents of FAD/Ti_3_C_2_T_x_/GCE were increased linearly with respect to the scan rate. From this observation, the redox potential remained the same and the peak currents were increased due to the surface-controlled process on the FAD/Ti_3_C_2_T_x_/GCE [18]. The ΔEp for FAD redox peak was found to be 16 mV, which suggested that immobilized FAD had a higher rate of electron transfer compared to the other modified electrodes [48]. Figure 7B shows the linear plot of ν vs. cathodic peak currents (I_pc_), which gave a straight line with the linear regression equation of Y = 4.37 × 10^−5^ + 8.08 × 10^−7^ (R^2^ = 0.999) [46]. The surface coverage of the FAD/Ti_3_C_2_T_x_ modified electrode was calculated using Equation (3)

(3) Ip=n2F2νAΓ4RT

where *n* is the number of electrons involved in the reaction (2), *F* is the faraday constant, *R* is the gas constant, *A* is the area of the electrode (0.13 cm^2^) and *T* is the temperature. From the slope of the linear curve, the surface coverage (ᴦ*_FAD_*) of the electrode was calculated as 0.8 × 10^−10^ mol/cm^2^ using Equation (3). This was similar to the surface coverage, which was calculated from Equation (1) as 0.7 × 10^−10^ mol/cm^2^. These values indicated the formation of the FAD monolayer (adsorption) on the surface of the Ti_3_C_2_T_x_ modified GCE.

### 3.8. Analysis of H_2_O_2_ Using FAD/Ti_3_C_2_T_x_/GCE

**Figure 8:**
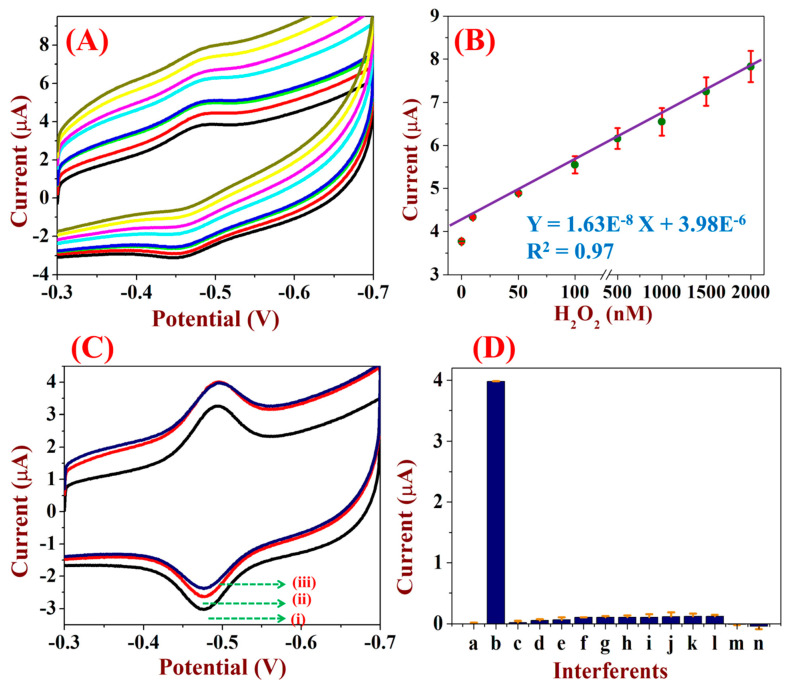
(**A**) CVs of FAD/Ti_3_C_2_T_x_/GCE were recorded in 0.1 M PBS with different concentrations of H_2_O_2_ from 5 nM to 2 µM at a scan rate of 50 mV/s. (**B**) The calibration plot was established between the concentrations of H_2_O_2_ and the reduction peak currents. (**C**) CVs were recorded using FAD/Ti_3_C_2_T_x_ modified electrode in 0.1 M PBS before (curve i) and after addition of 100 nM H_2_O_2_ (curve ii) and (curve iii) represents CV curve after the addition of 1 µM concentration of each interfering molecule with 100 nM H_2_O_2_. (**D**) The bar diagram represents the changes in the current responses caused by FAD/Ti_3_C_2_T_x_/GCE with (a) 0 nM H_2_O_2_ and (b) 100 nM H_2_O_2_. The catalytic current was recorded after the addition of 1 µΜ of each interfering molecule, (c) L-alanine, (d) L-isoleucine, (e) L-cysteine, (f) NaCl, (g) KCl, (h) glucose, (i) lactose, (j) AA, (k) UA, (l) OA (m) PA, and (n) DA (these experiments were repeated three times and the SD values are given, *n* = 3).

The FAD/Ti_3_C_2_T_x_/GCE was used as an electron transfer mediator for the detection of H_2_O_2_. CVs were recorded with the different concentrations of H_2_O_2_ from 5 nM to 2 µM using the FAD/Ti_3_C_2_T_x_/GCE (Figure 8A). It can be seen that the reduction peak currents were increased with the increment of H_2_O_2_ concentrations. A calibration plot was established between the [H_2_O_2_] and the reduction peak currents, which exhibited a linear regression equation of Y = 1.63 × 10^−8^ + 3.98 × 10^−6^ (R^2^ = 0.97) (Figure 8B) [49]. From the calibration curve of H_2_O_2_, the LOD was calculated as 0.7 nM using the formula below [50]:

LOD = 3.3 × standard deviation of the blank/slope of the calibration curve [51].

The obtained LOD was compared with the other reported methods using FAD as an electron transfer mediator. Previously, HA/HNT film was used for the immobilization of FAD on GCE and it was used for the analysis of H_2_O_2_ from 1 to 250 µM with a LOD of 0.49 µM [16]. Similarly, FAD immobilized on the carbon nanotube/chitosan modified electrode was used to detect H_2_O_2_ from 1 µM to 2.2 mM [17]. It was possible that FAD/Ti_3_C_2_T_x_/GCE could show better performance than the previously reported methods for H_2_O_2_ sensing, with improved sensitivity.

### 3.9. Selectivity of the FAD/Ti_3_C_2_T_x_/GCE Based Sensor

Selectivity is an important factor of the electrochemical sensors. The selectivity of the FAD/Ti_3_C_2_T_x_/GCE towards H_2_O_2_ was confirmed by CVs recorded with biomolecules associated with the human body. First, Figure 8D(a) CVs were recorded using FAD/Ti_3_C_2_T_x_/GCE with (b) 100 nM H_2_O_2_. This was followed by 10 fold concentrations of interfering substances being added, including ((c) L-alanine, (d) L-isoleucine, (e) L-cysteine, (f) NaCl, (g) KCl, (h) glucose, (i) lactose, (j) AA, (k) UA, (l) OA (m) PA, and (n) DA); then, current responses were recorded [52]. Figure 8C shows the CVs recorded for the FAD/Ti_3_C_2_T_x_/GCE in 0.1 M PBS before (curve-i) and after the addition of 100 nM H_2_O_2_, which showed that the reduction peak current was increased (curve-ii). Next, CVs were recorded with the subsequent additions of interfering molecules where the reduction current was significantly decreased (curve-iii). This analysis confirmed that the FAD/Ti_3_C_2_T_x_/GCE was more selective and responded only for H_2_O_2_, and not for the other biomolecules (Figure 8C). After analysis with the interfering molecules, the FAD reduction peak current was significantly decreased. Figure 8D shows the bar diagram of the overall current responses observed for the interfering substances. It was obvious that the selectivity of the FAD/Ti_3_C_2_T_x_/GCE towards H_2_O_2_ was maintained at about 98%.

The electro-catalytic reduction of H_2_O_2_ on FAD/Ti_3_C_2_T_x_/GCE was mediated by the FAD. The electrochemically reduced FAD to FADH_2_ by transfer of two electrons and protons. The reduced FADH_2_ aids in the reduction of H_2_O_2_ to H_2_O (see Equations (4) and (5)) [17,53].

(4) $$Ti_{3}C_{2}T_{x}-FAD+2H^{+}+2e^{-}\;↔Ti_{3}C_{2}T_{x\;}-FADH_{2}$$

(5) $$Ti_{3}C_{2}T_{x}-FADH_{2}+H_{2}O_{2}\;\to Ti_{3}C_{2}T_{x}-FAD+2\;H_{2}O$$

### 3.10. Stability, Reproducibility and Repeatability

**Figure 9:**
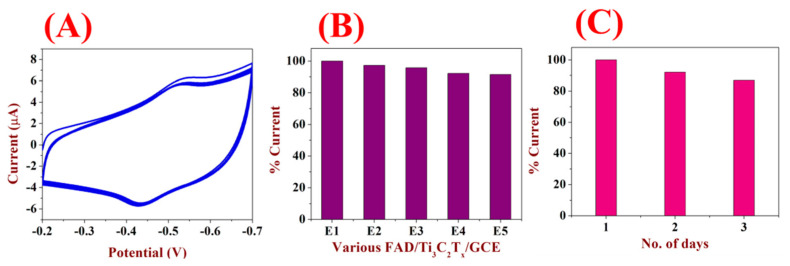
(**A**) CVs were recorded using the FAD/Ti_3_C_2_T_x_/GCE in 0.1 M PBS for the 50 continuous potential cycles at a scan rate of 50 mV/s. (**B**) The reproducibility of the five different FAD/Ti_3_C_2_T_x_/GCE’s were tested in 0.1 M PBS and the corresponding reduction current percentages were noted. (**C**) The repeatability of the FAD/Ti_3_C_2_T_x_/GCE was studied for continuous usage for 3 days in 0.1 M PBS and the changes in the reduction peak currents were recorded.

To study the stability of FAD/Ti_3_C_2_T_x_/GCE, CVs were recorded for 50 potential cycles in 0.1 M PBS at a scan rate of 50 mV/s. The redox peak currents of FAD were decreased by about 2% only after the 50 potential cycles (Figure 9A). The reproducibility of the FAD/Ti_3_C_2_T_x_-modified GCE was evaluated. For this purpose, five different FAD/Ti_3_C_2_T_x_/GCE’s were prepared as given in Section 2.3. The bar diagram represents the reduction peak currents of FAD/Ti_3_C_2_T_x_/GCE as shown in Figure 9B. The relative standard deviation (RSD) was 1.7%, which indicated that the described electrode fabrication procedure was accurate. The repeatability test was also studied by using FAD/Ti_3_C_2_T_x_/GCE for a period of three days, and the sensor stability was maintained up to 87% (Figure 9C).

### 3.11. ORR Study

**Figure 10:**
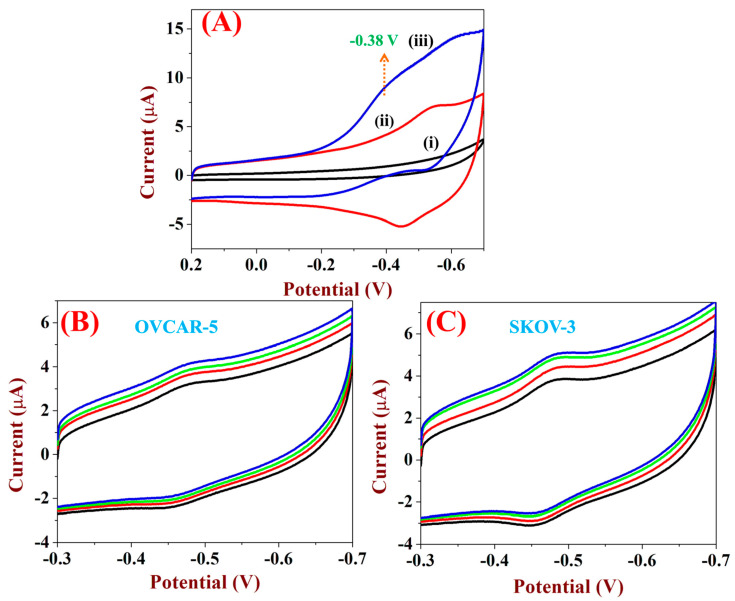
(**A**) The ORR on (curve i) bare GCE and (curve iii) FAD/Ti_3_C_2_T_x_/GCE in 0.1 M PBS with dissolved oxygen. Curve ii shows the CVs of the FAD/Ti_3_C_2_T_x_/GCE in N_2_ saturated solution, scan rate = 50 mV/s. (**B**,**C**) The CVs were recorded for the analysis of spiked H_2_O_2_ concentrations (10, 50 and 100 nM) in OVCAR-5 and SKOV-3 cancer cell lines samples using FAD/Ti_3_C_2_T_x_/GCE.

The oxygen reduction reaction (ORR) was also carried out on FAD/Ti_3_C_2_T_x_/GCE in O_2_ and N_2_ saturated PBS solution. When a bare GCE was used for the O_2_ reduction, there was no significant catalytic effect noted (Figure 10A, black curve i). However, for FAD/Ti_3_C_2_T_x_/GCE, the O_2_ reduction peak appeared at −0.38 V (curve iii). After the electrolyte solution was purged with N_2_, the redox peak of FAD appeared (curve ii). As shown in Figure 2C, the H_2_O_2_ reduction appeared at −0.47 V. This indicated that the FAD/Ti_3_C_2_T_x_/GCE could separate the reduction peak of H_2_O_2_ from the O_2_ reduction peak at different potentials. Thus, this sensor may be suitable for the selective detection of H_2_O_2_ in the presence of oxygen.

### 3.12. Detecting H_2_O_2_ in Ovarian Cancer Cell Lines

The real applications of the FAD/Ti_3_C_2_T_x_/GCE were tested in the analysis of H_2_O_2_ in OVCAR-5 and SKOV-3 ovarian cancer cell lines. As reported earlier, the excretion of H_2_O_2_ was higher in cancer cells [54]. The release of H_2_O_2_ has been reported in induction of cell death (PMID: 25433364; PMID: 27172875]. The cancer cells do not normally excrete H_2_O_2_, however, after adding the stimulant, they can release H_2_O_2_ by disrupted intracellular redox homeostasis of cancer cells [55]. For this purpose, AA was used as a stimulant. The FAD/Ti_3_C_2_T_x_/GCE was used to record CVs in 0.1 M PBS before and after the addition of the stimulant. Then, the OVCAR-5, SKOV-3 cell lines were added, which did not show any current responses. This indicated that the FAD/Ti_3_C_2_T_x_/GCE did not provide any response, which may be due to absence of H_2_O_2_ or the lower sensitivity of the sensor [56]. Using the proposed sensor, we confirmed that the ovarian cell lines did not excrete H_2_O_2_ while adding the stimulant. Furthermore, the known concentrations of H_2_O_2_ (such as 10, 50 and 100 nM H_2_O_2_) were spiked in the PBS containing the ovarian cancer cell lines. The cathodic peak currents were increased with respect to the spiked concentrations of H_2_O_2_ [57] (Figure 10B,C). From this analysis, the spiked concentrations of H_2_O_2_ were estimated and the recovery percentages (92–97.7%) were calculated (Table 1). These results corroborated that the FAD/Ti_3_C_2_T_x_/GCE may be used for the detection of H_2_O_2_ in cancer cell lines or other biological samples. However, further optimization and control studies will be required.

## 4. Conclusions

In summary, we have demonstrated that Ti_3_C_2_T_x_ is a biocompatible material for the immobilization of FAD by electrochemical deposition. The surface functional groups of Ti_3_C_2_T_x_ helped in the electro-adsorption of FAD. FAD-immobilized Ti_3_C_2_T_x_ was studied by UV-Vis and Raman spectroscopies, which confirmed the adsorption of FAD on the Ti_3_C_2_T_x_. The FAD-immobilized Ti_3_C_2_T_x_ electrode was used as an electrochemical transducer for the detection of H_2_O_2_. The amount of Ti_3_C_2_T_x_ and concentration of FAD were optimized to develop an effective sensor for H_2_O_2_. FAD/Ti_3_C_2_T_x_/GCE showed a redox peak at −0.1 V in 0.1 M H_2_SO_4_. Compared to the non-enzymatic sensors, Ti_3_C_2_T_x_-FAD modified electrode exhibited good sensitivity (0.125 µA nM/cm^2^) and a linear range of detection from 5 nM to 2 μM with an LOD of 0.7 nM. This FAD enzyme-modified electrode was very selective towards H_2_O_2_ reduction in the presence of other interfering molecules. Finally, the real application of the sensor was successfully demonstrated in the cancer cell lines samples spiked with known concentrations of H_2_O_2_, with good recovery.

## Figures and Tables

**Table 1:** The FAD/Ti_3_C_2_T_x_ modified electrode was used for the analysis of spiked H_2_O_2_ in to OVCAR-5 and SKOV-3 cancer cell lines.

Serial No.	Real Samples	Added (nM)	Found (nM)	Recovery (%)	RSD (%)
1.	OVCAR-5	10	9.77	97.7	1.72
		50	46.8	93.6	1.43
		100	93.2	93.2	2.36
2.	SKOV-3	10	9.43	94.3	1.25
		50	45.64	91.28	2.54
		100	91.9	92	3.17
